# Communicating the diagnosis of Klinefelter syndrome to children and adolescents: when, how, and who?

**DOI:** 10.1007/s12687-022-00585-0

**Published:** 2022-03-05

**Authors:** L. Aliberti, I. Gagliardi, S. Bigoni, S. Lupo, S. Caracciolo, A. Ferlini, A. M. Isidori, M. C. Zatelli, M. R. Ambrosio

**Affiliations:** 1 Department of Medical Sciences, Section of Endocrinology and Internal Medicine, University of Ferrara, Ferrara, Italy; 2 Unit of Medical Genetics, S. Anna University Hospital, Ferrara, Italy; 3 Endocrine Unit, Azienda Ospedaliero-Universitaria Di Ferrara, Cona/Ferrara, Italy; 4 Neurological, Psychiatric and Psychological Sciences Sections, Department of Biomedical and Specialty Surgical Sciences, School of Medicine, University of Ferrara, Ferrara, Italy; 5 Department of Experimental Medicine, Sapienza University of Rome, Rome, Italy

**Keywords:** Adolescents, Communication, Klinefelter syndrome, Disclosure, Multidisciplinary, Parents

## Abstract

Klinefelter syndrome (KS) is the most frequent sex chromosome aneuploidy in males. KS diagnosis disclosure has an important impact on diagnosis acceptance and the increase in prenatal diagnostic procedures raises questions regarding communication to children/adolescents. Limited data are currently available on this issue. The aim of the study was to investigate aspects like the best timing (when), topics (how), and healthcare professional (who), which, in the opinion of both KS patients and parents, may be considered the best for diagnosis communication to KS children/adolescents. We also analyzed how participants received the communication in real life and evaluated the differences between the responses given by parents who receive KS diagnosis before or after KS patient birth regarding disclosure of KS communication. KS adult patients, KS mothers, and KS fathers, not belonging to the same family, completed a questionnaire containing quantitative measures (5 points Likert scale), open-ended questions, and multiple choice questions. Parental responses were divided according to the timing at which the communication occurred: prenatal age diagnosis (PRE-D) or postnatal age diagnosis (POST-D). A total of 41 KS adults and 77 KS parents (53 PRE-D, 24 POST-D) were recruited. Most KS patients and most POST-D parents consider that communication should be provided before 14 years of age; most PRE-D parents consider 14–18 years of age the best period for communication. We suggest that communication should occur preferably before 18 years of age by a multidisciplinary team (endocrinologists, psychologists, geneticists, and parents) and that the information should deal not only fertility and hormonal aspects but also metabolic and cognitive features.

**Supplementary Information:**

The online version contains supplementary material available at 10.1007/s12687-022-00585-0.

## Introduction

Klinefelter syndrome (KS) is the most frequent sex chromosome aneuploidy in males with an estimated prevalence of 1/660 males and is caused by the presence of one extra X chromosome to a normal karyotype (47, XXY) (Radicioni et al. 2010). Even though KS phenotype is highly heterogeneous, the main features are tall stature, gynecomastia, decreased facial hair, hypergonadotropic hypogonadism, small testis, and infertility (Bearelly and Oates 2019; Davis et al. 2016; Radicioni et al 2010; Visootsak and Graham Jr 2006). During postnatal life, diagnosis can be made at different ages: in adulthood (25% of cases), often because of infertility, hypogonadism, or erectile dysfunction, or in childhood (more rarely and incidentally), because of cryptorchidism, hypospadias, speech delay, learning disorders, gynecomastia, and delayed puberty (Bird and Hurren 2016; Boada et al. 2009; Bonomi et al. 2017; Tartaglia et al. 2008; Zitzmann et al. 2004). Many individuals living with this condition, however, remain undiagnosed (Bojesen et al. 2003). Moreover, an increasing proportion of KS diagnoses occurs during prenatal cytogenetic testing by means of karyotype analysis (Bearelly and Oates 2019; Davis et al. 2016; Radicioni et al 2010; Tartaglia et al. 2008; Visootsak and Graham Jr 2006).

Healthcare professionals (HCPs) play a critical role in diagnosis communication in all fields of medicine and those dealing with KS should provide adequate counseling (Bancroft et al. 1982; Bourke et al. 2014; Gies et al. 2014; Tremblay et al. 2016; Zitzmann et al. 2021). Furthermore, when a chromosomal aneuploidy is diagnosed during prenatal age/childhood, parents often face the decision of “how and when” to disclose the diagnosis to their child. Few data are currently available in the literature on this issue and more evidence is needed to establish best practices (Suwannachat et al. 2020; Tremblay et al. 2016). Understanding at which age communication should occur would be useful since it may influence diagnosis acceptance. In the view of unaffected adolescent males, late disclosure may involve such disadvantages as greater difficulty in diagnosis acceptance (Suwannachat et al. 2020). Previous evidence reports that age of diagnosis communication negatively correlates with adaptation, suggesting that older participants experience more challenges throughout their lives compared to younger subjects (Turriff et al. 2015). Common concerns among KS parents include disclose timing, uncertainty about what words to use or how to discuss this issue in relation to age (Dennis et al. 2015). In addition, KS parents worry that the child may not understand or may react negatively to the diagnosis and become emotional during the conversation (Dennis et al. 2015). Moreover, the greater the availability of opinions expressed by KS parents and patients could help HCPs to promptly identify opportunities for intervention to promote quality of life and adaptation (Turriff et al. 2015). For instance, issues with body image, self-esteem, or bullying may be more relevant among adolescents and young adults, while infertility may raise more concerns among people who desire to start families (Turriff et al. 2015).

Parents may have different opinions about the age at which their son is developmentally ready to learn about his diagnosis. Some parents decide to disclose the diagnosis as soon as their son begins to ask questions related to his health. Others, instead, adopt the “seed-planting” strategy, which consists of an early disclosure at 3–4 years of age followed by subsequent conversations so that the child can continuously develop his understanding (Dennis et al. 2015; Gratton et al. 2016; Holmes-Siedle et al. 1987; Robinson et al. 1989; Suwannachat et al.^2020^). While some parents prefer to disclose information related to the syndrome gradually, others choose to wait for the “right time” in their son’s life (Close et al. 2016; Dennis et al. 2015; Mac Dougall et al. 2007; Gratton et al. 2016; Metcalfe et al. 2008; Turriff et al. 2017). Lastly, some parents may indefinitely postpone diagnosis disclosure (often fearing a negative impact on self-esteem, risk of stigmatization, discrimination, and bullying) (Close et al. 2016; Mac Dougall et al. 2007; Metcalfe et al. 2008; Turriff et al. 2017). Metcalfe et al. (2011) reported that in families where parents communicated the genetic condition in childhood, affected patients coped well with the condition, whereas in families who tried to keep KS information secret, children had increased stress and negative emotional experiences.

For children with Turner syndrome (TS), discussions on diagnosis and treatment are recommended as soon as the child is able to understand (Frías et al. 2003; Tremblay et al. 2016), whereas for KS, no indications have so far been stated by scientific societies concerning either the best patient age for communication, or the HCP most suited for the task or which topics should be discussed. As previously stated, few data are present on KS communication to children/adolescents and more evidence is needed for best practices.

The aim of our study was to investigate the opinion of KS patients and KS parents concerning the best timing (when), which topics (how) and the most suitable HCP to communicate the diagnosis to KS children/adolescents as well as individual opinions on emotions generated among the listeners (teachers, friends, schoolmates) by sharing the diagnosis. We also analyzed how KS adult patients and KS parents received communication of the genetic diagnosis in real life (in particular, topics communicated during disclosure), and then we evaluated the differences between the answers of parents who receive KS diagnosis before or after KS patient birth regarding disclosure of KS communication.

## Material and methods

### Participants

Individuals aged 18 years old and older with blood karyotype 47, XXY, and KS mothers/fathers were recruited during endocrinological examinations and/or through the support of the “Nascere Klinefelter” Association.

### Procedures

Participants were invited to complete a self-administered and anonymous questionnaire based on open-ended questions, multiple-choice questions, and statements based on quantitative measures (See Supplementary material). There was no financial compensation for the participants. This study was approved by the local ethic committee. Informed consent was obtained from all patients. The questionnaire was edited on the basis of published evidence concerning the communication of diagnosis of sex aneuploidies (Dennis et al. 2015; Turriff et al. 2017) and included questions/statements about the following:

–demographic features
–the timing of diagnosis communication
–topics discussed during diagnosis communication
–HCPs who communicated the diagnosis
–individual opinions on when and how the diagnosis should be disclosed and who should do it
–individual opinions on emotions generated among the listeners (teachers, friends, schoolmates) by sharing diagnosis

### Data analysis

The survey instrument included a questionnaire containing open-ended questions, multiple choice questions, and statements based on quantitative measures (from 1 to 5 in accordance with a 5-point Likert scale) (Dalkey 1969). For multiple-choice questions, answers were identified and frequencies were calculated. For statements based on quantitative measures (from 1 to 5 in accordance with a 5-point Likert scale), answers 1 and 2 (disagreement) and answers 3, 4, and 5 (agreement) were merged according to the Delphi method, in particular as follows:

–1: absolutely disagree
–2: disagree
–3: agree
–4: more than agree
–5: absolutely agree

The Delphi method aims at reaching the best estimate of consensus and at providing recommendations on controversial topics (Dalkey 1969). Consensus was reached when the sum of items 1 and 2 (disagree) or 3, 4, and 5 (agree) reached 66%. Where no consensus was reached, the results were shown as neither disagree/nor agree (ND/NA), with ND standing for the sum of items 1 and 2, and NA as the sum of items 3, 4, and 5. Parents’ answers were split according to the timing at which the genetic diagnosis was communicated, i.e., prenatal age diagnosis (PRE-D) and postnatal age diagnosis (POST-D), since they may have had different opinions concerning KS communication (Metcalfe et al. 2011). Moreover, within each group (PRE-D and POST-D), mothers’ and fathers’ answers were analyzed separately. Comparison of qualitative variables was performed by means of the Fisher’s exact test. *P* values < 0.05 were considered as indicating statistical significance.

## Results

A total of 41 KS adult patients and 77 KS parents, all Caucasians, completed the survey. In particular, 53 PRE-D (43 mothers and 10 fathers) and 24 POST-D parents (19 mothers and 5 fathers) were recruited into the study. KS patients and parents did not belong to the same families. Participants were asked to recall events in the past. PRE-D and POST-D parents received KS diagnosis information 14.7 ± 6.9 years and 18.5 ± 7.1 years, respectively, before participating in the survey. Moreover, KS patients had received diagnosis on average 15.4 ± 6.3 years before this study. Participant characteristics are summarized in Table 1. HCPs involved in diagnosis communication are summarized in Table 2. In real life, most of KS adult patients received the diagnosis during adult age (93%) and only 3 patients (7%) received diagnosis when they were < 18 years old.

**Table 1:** General characteristic of participants

		Prenatal age diagnosis (PRE-D)		Postnatal age diagnosis (POST-D)	
	KS Patients	Mothers	Fathers	Mothers	Fathers
Participants *N* (%)	41	43	10	19	5
Age (years): mean ± SD	43.4 ± 11.3	43.1 ± 7.6	42.2 ± 3.4	48.32 ± 9.65	46 ± 13.06
Age range (years)	20–76	35–51	39–46	25–68	25–59
Elementary/junior high school *N* (%)	12/41 (29.3%)	4/43 (9.3%)	0/10 (0%)	4/19 (21.1%)	1/5 (20%)
High school *N* (%)	27/41 (65.9%)	23/43 (53.5%)	2/10 (20%)	11/19 (57.9%)	3/5 (60%)
University *N* (%)	2/41 (4.9%)	16/43 (37.2%)	8/10 (80%)	4/19 (21.1%)	1/5 (20%)

**Table 2:** HCPs who communicated diagnosis

		Prenatal age diagnosis (PRE-D)		Postnatal age diagnosis (POST-D)	
HCPs who communicated diagnosis:	KS patients *N* (%)	Mothers *N* (%)	Fathers *N* (%)	Mothers *N* (%)	Fathers *N* (%)
Geneticist	9/41 (22%)	31/43 (72%)	6/10 (60%)	9/19 (47.4%)	3/5 (60%)
Gynecologist	1/41 (2.4%)	12/43 (28%)	4/10 ( 40%)	/	/
Endocrinologist	20/41 (48.8%)	/	/	6/19 (31.6%)	1/5 (20%)
Pediatrician	/	/	/	2/19 (10.5%)	1/5 (20%)
Urologist	5/41 (12.2%)	/	/	/	/
General practitioner	5/41 (12.2%)	/	/	/	/
Non-responders	1/41 (2.4%)	/	/	2/19 (10.5%)	/

### Best age for diagnosis communication

Most of the KS adult patients believed that diagnosis communication should take place before 14 years old (53.7%) or between 14 and 18 years of age (39%) (Table 3). Most of the PRE-D mothers declared that diagnosis should be communicated between 14 and 18 years of age (44.2%), whereas 34.9% of them stated that the best age for communication should be before the child’s 14th year. POST-D mothers would communicate KS diagnosis before 14 years of age in 73.7% of cases and none would communicate diagnosis after 18 years of age (Table 3). The distribution of answers between PRE-D and POST-D mothers is significantly different (*p* < 0.05).

**Table 3:** Best age for communication of diagnosis according to the opinion of participants

Responders	Best age for communication of diagnosis:			
	< 14 yrs	14–18 yrs	> 18 yrs	NR
KS patients ^a^ Answers *N* (%)	22 (53.7%)	16 (39%)	1 (2.4%)	2 (4.9%)
PRE-D mothers ^b^ Answers *N* (%)	15 (34.9%)	19 (44.2%)	8 (18.6%)	1 (2.3%)
PRE-D fathers ^c^ Answers *N* (%)	1 (10%)	5 (50%)	3/10 (30%)	1 (10%)
POST-D mothers ^b^ Answers *N* (%)	14 (73.7%)	5 (26.3%)	0%	0%
POST-D fathers ^c^ Answers *N* (%)	5 (100%)	0%	0%	0%

*PRE-D*, prenatal age diagnosis; *POST-D*, postnatal age diagnosis; *KS*, Klinefelter syndrome; *NR*, non-responders; *Yrs*, years old

^a^*p* < 0.05 KS vs. PRE-D parents’ answers

^b^*p* < 0.05 PRE-D vs. POST-D mothers’ answers

^c^*p* < 0.01 PRE-D vs. POST-D fathers’ answers

Most of the PRE-D fathers indicated that the diagnosis should be communicated when the patient is 14–18 years old (50%) or after 18 years of age (30%). All POST-D fathers would communicate diagnosis before 14 years of age and none after 18 years of age (Table 3). The distribution of answers between PRE-D and POST-D fathers is significantly different (*p* < 0.01). Moreover, the distribution of answers between KS patients and PRE-D parents’ answers is significantly different (*p* < 0.05).

### Topics discussed during the diagnosis communication and emotions generated by sharing diagnosis

KS adult subjects agreed that fertility and hormonal issues were described during the communication (consensus reached), whereas cognitive and metabolic syndrome topics were not fully addressed (ND/NA). PRE-D and POST-D mothers/fathers agreed they received adequate information about fertility, cognitive, and hormonal features (consensus reached), whereas metabolic issues were not completely addressed (ND/NA). Topics discussed during diagnosis communication are summarized in Table 4.

**Table 4:** Issues treated during communication of diagnosis

		Prenatal age diagnosis (PRE-D)		Postnatal age diagnosis (POST-D)	
	Total KS patients (*N* 41)	Mothers (*N* 43)	Fathers (*N* 10)	Mothers (*N* 19)	Fathers (*N* 5)
Issues treated	Sum of answers 3, 4, and 5 on the Likert scale: *N* (%)	Sum of answers 3, 4, and 5 on the Likert scale: *N* (%)	Sum of answers 3, 4, and 5 on the Likert scale: *N* (%)	Sum of answers 3, 4, and 5 on the Likert scale: *N* (%)	Sum of answers 3, 4, and 5 on the Likert scale: N°(%)
Fertility	31/41 (76%) NR: 2/41 (5%)	38/43 (88%) NR: 0	9/10 (90%) NR: 0	15/19 (79%) NR: 1/19 (5%)	4/5 (80%) NR: 0
Metabolic features	16/41 (39%) NR: 2/41 (5%)	28/43 (65%) NR: 0	6/10 (60%) NR: 0	10/19 (53%) NR: 1/19 (5%)	3/5 (60%) NR: 0
Cognitive features	20/41 (49%) NR: 1/41 (2.4%)	33/43 (77%) NR: 0	9/10 (90%) NR: 0	13/19 (68%) NR: 1/19 (5%)	4/5 (80%) NR: 0
Hormonal features	30/41 (73%) NR: 1/41 (2.4%)	32/43 (74%) NR: 0	9/10 (90%) NR: 0	13/19 (68%) NR: 0	4/5 (80%) NR: 0

*NR*, non-responders

PRE-D mothers/fathers think that sharing the diagnosis with friends, schoolmates, and teachers generates fear (ND/NA), pity (ND/NA), and misunderstanding (ND/NA) but not empathy (agreement). POST-D mothers/fathers and KS adult patients disagree that sharing diagnosis creates fear, pity, and misunderstanding (consensus reached). Moreover, they agree that sharing the diagnosis causes empathy among the listeners (consensus reached). Opinions of participants are summarized in Table 5.

**Table 5:** Emotions generated among the listeners (teachers, schoolmates, friends) by sharing diagnosis

		Prenatal age diagnosis (PRE-D)		Postnatal age diagnosis (POST-D)	
	Total KS Patients (*N* 41)	Mothers (*N* 43)	Fathers (*N* 10)	Mothers (*N* 19)	Fathers (*N* 5)
Emotions generated among the listeners	Sum of answers 3, 4, and 5 on the Likert scale: *N *(%)	Sum of answers 3, 4, and 5 on the Likert scale: *N *(%)	Sum of answers 3, 4, and 5 on the Likert scale: *N *(%)	Sum of answers 3, 4, and 5 on the Likert scale: *N *(%)	Sum of answers 3, 4, and 5 on the Likert scale: *N *(%)
Empathy	29/41 (71%) NR: 0	14/43 (32%) NR: 0	3/10 (30%) NR: 0	13/19 (68%) NR: 0	4/5 (80%) NR: 0
Pity	13/41 (32%) NR: 0	23/43 (53%) NR: 0	6/10 (60%) NR:0	4/19 (21%) NR: 0	0/5 (0%) NR: 0
Fear	12/41 (29%) NR: 1/41 (2.4%)	27/43 (63%) NR: 0	6/10 (60%) NR: 0	5/19 (26%) NR: 0	0/5 (0%) NR: 0
Misunderstanding	12/41 (29%) NR: 0	23/43 (53%) NR: 0	5/10 (50%) NR: 0	3/19 (16%) NR: 0	1/5 (20%) NR: 0

*NR*, non-responders

### Who should communicate the diagnosis?

**Fig. 1:**
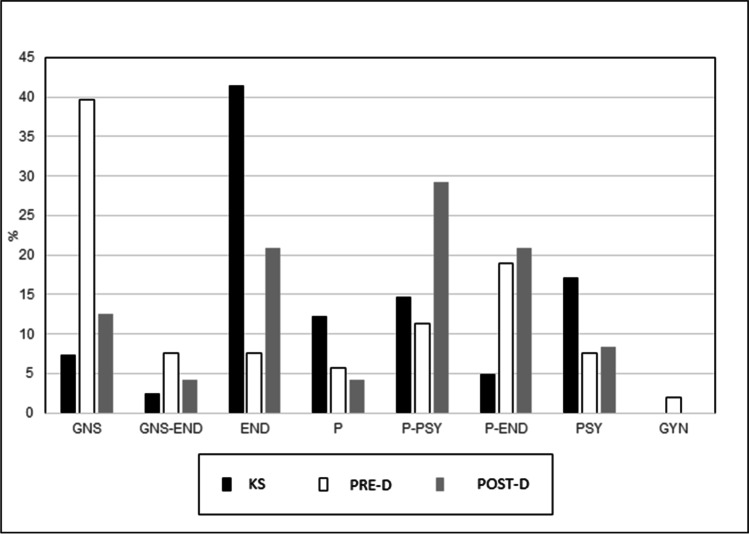
HCPs/person who should communicate diagnosis to KS children/adolescents. GNS, geneticists; GNS-END, geneticists together with endocrinologists; END, endocrinologists; P, parents; P-END, parents together with endocrinologists; P-PSI, parents together with psychologists; PSY, psychologists; GYN, gynecologists; PRE-D, prenatal diagnosis; POST-D, postnatal diagnosis

Almost half of KS adult patients (41.5%) would like the diagnosis to be communicated by an endocrinologist; a lower percentage reported that it should be communicated by a psychologist (17%) or by parents alone (12.2%). In the opinion of PRE-D parents, diagnosis should be communicated mostly by geneticists (39.6%) or by parents together with endocrinologists (18.9%). POST-D parents indicated that diagnosis should be communicated by parents together with a psychologist (29%), by parents alone (20.9%), or by parents together with endocrinologists (20.9%) (Fig. 1).

## Discussion

Our study explores the opinion of KS patients and KS parents concerning the best timing (when), topics (how), and HCP who should communicate the diagnosis to KS children/adolescents. Our results show that most PRE-D mothers and fathers prefer KS diagnosis communication at 14–18 years of age, while most KS patients and POST-D parents indicate that the best timing for KS diagnosis communication is before 14 years of age. Concerning the HCP in charge of diagnosis communication, heterogeneous answers were expressed, suggesting the need for a multidisciplinary team for this purpose. Indeed, there is agreement in the literature on the importance and value of the multidisciplinary team as a means of providing care for KS patients (Groth et al. 2013).

The increase in prenatal diagnoses highlights the problem of KS diagnosis communication to children and adolescents. So far, there are limited data in the literature on this issue and few indications that HCPs and parents can follow. To our knowledge, this is the first study that investigates the opinions of KS adult patients about “when” KS diagnosis communication to children/adolescents should take place and “who” should do it. Differently from other reports, the quantitative measures of the answers allowed a statistical analysis of the participants’ opinions (Dennis et al. 2015; Tremblay et al. 2016; Turriff et al, 2017). Very few studies have previously investigated the issue of KS diagnosis communication, and their subject group included only KS parents or very few adult KS patients (Suwannachat et al. 2020) or individuals with other chromosome aneuploidies and did not provide quantitative evaluations (Dennis et al. 2015). Indeed, Hanna et al (2019) reported that the views of KS patients appear to be lacking from the literature and that future research should attempt to capture the real-life experience of KS patients.

Disclosure of diagnosis plays an important role within illness acceptance of KS syndrome, quality of life, and the patient’s psychological health. Unlike KS adults (who usually have more self-awareness), diagnosis communication during childhood/adolescence is a more delicate process, which could generate frustration and anxiety (Heshka et al. 2008; La Pean and Farrell 2005; Rosas-Blum et al. 2007).

Appropriate initial messages are important for effective communication because they form a foundation for comprehension of subsequent messages, and if they are not communicated well can lead to misunderstandings that contribute to general distress, greater anxiety, feelings of guilt, distorted self-images, and misinformed decisions (Heshka et al. 2008; La Pean and Farrell 2005; Rosas-Blum et al. 2007).

Nowadays, families adopt multiple lines of conduct (Close et al. 2016; Dennis et al. 2015; Mac Dougall et al. 2007; Gratton et al. 2016; Metcalfe et al. 2008; Turriff et al. 2017). Some participants did not respond to all the questions and we do not know whether this was due to difficulty in interpreting the text or to an unwillingness to respond. More mothers than fathers responded to the questions suggesting that mothers are more interested in the theme of communication as compared to fathers. Indeed, in papers regarding KS communication to children, there is a preponderance of mothers’ responses (Dennis et al 2015; Suwannachat et al 2020). It has previously been reported that mothers show greater interest and attendance and are overall more actively engaged with the HCP in their child’s intervention also in other medical conditions (DeMarco et al. 2008; Zaidman-Zait et al. 2018). Moreover, we split mothers and fathers’ answers because, as reported for other conditions (i.e., cystic fibrous carrier testing), men and women tend to differ in terms of their perception of risk, negative psychological effect, and perceptions about themselves (Newman et al. 2002). Moreover, parents may be separated so there may be conflicting relationships and different opinions about KS communication (DeMarco et al. 2008; Newman et al. 2002; Zaidman-Zait et al. 2018).

Our analysis found that most POST-D mothers/fathers and KS patients indicate that the best timing for KS diagnosis communication is before 14 years of age while most PRE-D mothers/fathers prefer KS diagnosis communication at 14–18 years of age. Indeed, families with postnatal diagnosis often discovered KS subsequent to investigations for their child’s health problems. Therefore, they may prefer an earlier diagnosis communication to their child to alleviate the patients’ concerns about having “something wrong” (Dennis et al. 2015; Linden et al., 2002; Sutton et al. 2006). Children often become aware of the situation and become stressed (Dennis et al. 2015; Linden et al., 2002; Sutton et al. 2006). An atmosphere of secrecy might also discourage children from asking questions (Metcalfe et al. 2011). Children between 9 and 15 years are able to differentiate between biological inheritance and cultural transmission (Solomon et al., 1996; Venville et al. 2005). However, we did not investigate children’s developmental stages and knowledge of kinship, issues that deserve further exploration. Finally, most KS adults and POST-D parents may prefer earlier communication also because this approach possibly facilitates better compliance with hormonal supplementation/clinical evaluations and a better quality of life (Dennis et al. 2015).

A recent study performed in Thailand showed that 4 KS adults and 8/14 PRE-D parents preferred communication to occur during the early teens or before hormonal therapy, but the level of maturity of children in Asian countries may differ from most Western countries due to cultural and societal differences (Suwannachat et al.^2020^).

In our study, KS adult patients agreed they received adequate information about fertility and hormonal features, whereas cognitive and metabolic issues were not well addressed. KS adult subjects often found out about their genetic condition is subsequent to investigations for infertility and it is likely that the HCPs who communicated the diagnosis mostly focused on fertility issues. Another hypothesis is that HCPs were not familiar with other aspects of KS and therefore did not deal with these issues. Indeed, in almost 1/3 of KS cases, the diagnosis was provided by urologists, gynecologists, or general practitioners (GPs), all HCPs who do not frequently deal with KS. In a study by Bourke et al. (2013), KS parents reported that HCPs, including GPs, pediatricians, obstetricians, and neurologists, provided information indicating that their knowledge of KS treatment and management was not up-to-date.

In our study, PRE-D mothers/fathers and POST-D mothers/fathers received adequate information about fertility, cognitive, and hormonal features (consensus reached); metabolic issues were not fully addressed (NA/ND). In these cases, the diagnosis was communicated mostly by geneticists (for PRE-D parents) and by endocrinologists (for POST-D parents), indicating that these HCPs are more familiar with the various aspects of KS syndrome. The theme of fertility was the principal issue explained, most likely because it is the characteristic sign of KS and clinicians may consider it to be the key feature to communicate. Indeed, Dennis and colleagues showed that the most common parental concerns were related to fertility issues and their impact on a child’s self-esteem (Dennis et al. 2015). Infertility was reported as one of the greatest challenges faced by KS patients (Turriff et al. 2017). Sutton et al. (2006) reported that disclosing infertility to TS patients was especially challenging for parents. Indeed, infertility comes with the added burden of existing social stigma, and the social emphasis placed on childbearing contributes to society’s perception that infertility is an abnormal condition, counter to societal expectations and norms (Sutton et al 2006; Whiteford & Gonzalez 1995). As for TS, an infertility diagnosis demands early and full disclosure because it may cause identity crises; disclosing diagnosis at a young age may enable patients to create self-images which do not include biological fatherhood (Lalos 1999; Sutton et al 2006; Whiteford & Gonzalez 1995). Moreover, our study suggests that communication concerning metabolic and cognitive issues related to KS deserves more attention, according to the opinions of KS patients.

There was a wide variety of responses about who should communicate the diagnosis. In our study, most PRE-D mothers/fathers believe that the diagnosis should be communicated by geneticists, owing, perhaps, to the good experience of counseling they had had during pregnancy that had led them not to consider abortion. In our study, most POST-D mothers consider that communication should be performed by parents together with psychologists. Finally, in our study, most KS patients and POST-D fathers want endocrinologists to communicate KS, and we hypothesized that this outcome may depend on the fact that they were satisfied with the endocrinologists following them. Therefore, our results and our center’s experience suggest that disclosure to KS children/adolescents may be provided by a team composed of parents collaborating with HCPs (an endocrinologist, geneticist, and psychologist). In particular, the role of the psychologist is to address emotional/psychological aspects of KS, whereas clinicians are fundamental for providing the correct health information on KS. Suwannachat et al. (2020) suggested that diagnosis should be communicated only by parents, but these authors investigated opinions of unaffected adolescent males and KS parents and considered only 4 KS adult subjects (Suwannachat et al.^2020^). Moreover, the study by Suwannachat was conducted among Thai participants; therefore, cultural and social differences may have influenced parental opinions (Suwannachat et al.^2020^).

Finally, our study is the first to investigate what participants thought about emotions generated by sharing genetic conditions with teachers, friends, and schoolmates. POST-D parents and KS patients disagreed that talking about KS elicits fear and pity among the listeners and agreed that the sharing of diagnosis may generate empathy among the listeners (consensus reached). In contrast, PRE-D families neither agreed nor disagreed that sharing the diagnosis could generate misunderstanding, fear, and pity among the listeners and disagreed that it may elicit empathy. Suwannachat et al. (2020) showed that PRE-D parents and unaffected adolescent males preferred keeping diagnosis confidential whereas KS adult participants referred mostly that the decision was up to the child (Suwannachat et al. 2020). PRE-D parents may consider KS diagnosis as a secret to be maintained, possibly to protect their child or themselves from bullying, stigma, pity, and prejudice (Close et al. 2016; Dennis et al. 2015; Mac Dougall et al. 2007; Gratton et al. 2016; Metcalfe et al. 2008; Turriff et al. 2017). Ashida et al. (2010) showed that sharing the genetic test results with friends was associated with a decrease in depression and anxiety levels. For BRAC1/2 test results, availability of social support was significantly associated with better psychological adjustment over time among carriers (Lapointe et al.^2013^). Tremblay et al. (2016) recommended encouraging parents to discuss KS with teachers. Informing teachers/friends might help both teachers (to reach a better understanding of their KS pupils, through “tailored” academic support) and schoolmates (to help and not to bully young KS subjects) (Tremblay et al. 2016). On the other hand, parents may fear bias on the part of the teachers’ belief in KS patients’ ability to obtain academic success, with the result that they feel discouraged to improve their performance.

## Limitations

A number of limitations affect our study. The sample size is not large and is composed only of Caucasian subjects with a high level of education and mostly of mothers with prenatal diagnosis. Moreover, there are few fathers and their level of education was high. We have a limited number of answers from KS patients that received diagnosis communication during adolescence/childhood. Survey responses are based only on participant reports and subject to recall bias (participants may have some difficulty remembering). The option “ < 14 years” for passing information to the child may be too broad and so it would be useful to split up this category into different sub-categories (i.e., age of children) to define better at which age an early disclosure should occur. In future, other questions might be added to our questionnaire aimed at filling in the gap in our understanding; these could include the age at which parents communicated diagnosis to their children in real life, emotions aroused by diagnosis communication (i.e., anger, relief, fear) among KS patients, and KS patients’ opinion (not only KS parents’ opinion) about the words to use/not to use during communication. In future researches, a combination of both qualitative and quantitative approach could be used for data collection and it might be useful to ask KS patients whether they would prefer the “seed planting strategy” or the “right time strategy” for diagnosis disclosure (Mac Dougall et al. 2007). Future studies should try to address the issues in which consensus was not reached (possibly through face-to-face discussion).

## Conclusions

Our study provides indications on how, when, and who should communicate the diagnosis to KS children/adolescents, as well as highlighting individual opinions on emotions generated among the listeners (teachers, friends, schoolmates) by sharing diagnosis.

Our conclusions report that communication should be performed before 14 years of age (according to most KS patients and POST-D mothers/fathers’ answers), whereas both PRE-D fathers and mothers believe that the best timing is between 14 and 18 years of age. However, the general preference is that communication should not occur after 18 years of age. Concerning the HCP in charge of diagnosis communication, the answers were heterogeneous, suggesting the need for a multidisciplinary team for this purpose. KS patients reported they did not receive information about metabolic and cognitive features. Clinicians should, therefore, provide information to KS patients not only on fertility and hormonal aspects but also on the aforementioned issues. Our study shows that POST-D parents and KS patients think that sharing diagnosis beyond the family does not generate fear and pity among listeners but empathy (Table 6).

**Table 6:** Disclosure of KS communication to children and adolescents

When	•Before 14 years of age (according to most KS patients and most POST-D mothers/fathers’ opinions) •Between 14 and 18 years of age (according to most PRE-D mothers/fathers’ opinions) •Preferably not after 18 years of age
Who	•A multidisciplinary team composed of parents and/or endocrinologists and/or psychologists and/or geneticists
How	Providing accurate information not only about fertility and hormonal aspects but also about metabolic and cognitive features
How	Stressing that POST-D parents and KS patients think that sharing diagnosis beyond family (with teachers, friends, schoolmates) does not generate fear and pity but empathy among listeners (teachers, schoolmates, friends)

## Supplementary Information

Below is the link to the electronic supplementary material.

Supplementary file1 (DOC 57 KB)

## Data Availability

Data that support the findings of this study are available on request from the corresponding author. The data are not publicly available due to privacy or ethical restrictions.
