# Supplementary material for: Communicating the diagnosis of Klinefelter syndrome to children and adolescents: when, how, and who?
Source: J Community Genet. 2022 Mar 5;13(3):271–80. doi: 10.1007/s12687-022-00585-0 (PMC9270507; doi:10.1007/s12687-022-00585-0)
Supplement: Supplementary file 1 — Supplementary file1 (DOC 57 KB) [file 12687_2022_585_MOESM1_ESM.doc]

**KLINEFELTER SINDROME (KS) DIAGNOSIS COMMUNICATION QUESTIONNAIRE**

- Which category do you belong to:

□ KS Patient □ KS mother □ KS father

- Level of education

□ Elementary □ Junior High School □High School □ University

- Age :_______________

- Race: □ Caucasian □ Asian □Afro- American □ Other:_____________________

**1.** At which age did you receive KS diagnosis communication? **(FOR PARENTS) :____________________(years)**

**2.** At which age did you receive KS diagnosis communication? **(FOR PATIENTS) :____________________(years)**

**3.** Which HCP communicated diagnosis?

□ Geneticist

□ Gynecologist

□ Endocrinologist

□ Pediatrician

□ Urologist

□ Psychologist

□ General Practitioner

□ Others____________________________________________________________________________________

**4.** Who do you think should communicate diagnosis?

□ Geneticist

□ Gynecologist

□ Endocrinologist

□ Pediatrician

□ Urologist

□ Psychologist

□ Parents

□ General Practitioner

□ Others____________________________________________________________________________________

**5.** During communication, the following features were explained well. Give a number from 1 to 5 *(1.Absolutely disagree; 2. Disagree; 3.Agree; 4. More than Agree; 5. Absolutely agree)*

**5A.** Fertility (testicular hypotrophy with reduction/absence of spermatogenesis)

1 2 3 4 5

□ □ □ □ □

□ Comments_________________________________________________________________________­­­­­­­­­­­­­­____________

**5B.** Metabolic features were explained (risk of developing metabolic syndrome, diabetes, abdominal adiposity, dyslipidemia, cardiovascular disease, etc.)

1 2 3 4 5

□ □ □ □ □

□ Comments___________________________________________________________________________________

**5C.** Hormonal consequences of hypogonadism were explained *(gynecomastia, decreased facial and pubic hair~~s~~, decreased penile size, decreased sexual desire, osteoporosis/osteopenia, reduction in lean mass, reduction in muscular strength, anemia, etc.)?*

1 2 3 4 5

□ □ □ □ □

□ Comments_____________________________________________________________________________________

**5D.** Cognitive aspects *(speech delay, learning abilities, psychosocial problems, etc )*

1 2 3 4 5

□ □ □ □ □

□ Comments____________________________________________________________________________________

**6.** Which is the best timing for disclosure of KS diagnosis? Give a number from 1 to 5 *(1.Absolutely disagree; 2. Disagree; 3.Agree; 4. More than Agree; 5. Absolutely agree)*

**6A.** Before 14 years of age

1 2 3 4 5

□ □ □ □ □

**6B.** Between 14-18 years of age

1 2 3 4 5

□ □ □ □ □

**6C.** After 18 years of age

1 2 3 4 5

□ □ □ □ □

**7.** Emotions generated among the listeners (teachers, friends, schoolmates) by sharing diagnosis. Give a number from 1 to 5 (*1.Absolutely disagree; 2. Disagree; 3.Agree; 4. More than Agree; 5. Absolutely agree)*

1 2 3 4 5

Pity □ □ □ □ □

Fear □ □ □ □ □

Misunderstanding □ □ □ □ □

Sympathy □ □ □ □ □
